# Porcelain Fillings

**Published:** 1902-02-15

**Authors:** W. C. Capon

**Affiliations:** Philadelphia Pa.


### PORCELAIN FILLINGS.

BY W. C. CAPON, D.D.S., PHILADELPHIA PA.

Extracts from a paper read at the New Jersey State Society.

Almost daily I am asked the question, "To what extent will I recommend that porcelain should be used?" I reply that much depends on one's adaptability to unite art with mechanics. In other words, to know when and where beauty and strength can be harmonized to a practical extent. Such a knowledge is desirable in general dentistry, but in a great degree requisite in the practice of this special branch.

My operations have extended over many hundreds of cases, and it is to be expected that our best results come from simple inlays. I mean by that term a cavity filled with porcelain on the labial or buccal surfaces of teeth where there is no strain or wear except that of ordinary cleanliness; yet it is a surprise to know the great durability of corner sections.

I have a number of cases ranging from eight to twelve years of service that I have watched with great interest, and in a few cases they have been positively abused, and yet they stand the strain although held with cement without the aid of pins or other means.

Porcelain is an ideal filling in approximal cavities. It saves poor structure and, to a great extent, conceals the fact that they are filled more than in many other positions. It is very gratifying to know that by this means I have been able to give a natural appearance to the teeth of many ladies who came to me as young girls with cement or discolored gutta-percha remnants and a source of satisfaction to these young patients, not to have the same cavities excavated and refilled every few months.

I have been alluding to the six anterior teeth because it is there we find the greater number of opportunities where porcelain is applicable. Next I shall mention the distal approximal surfaces of cuspids, especially when the cavity is extensive. In such places porcelain is an excellent material because it enables the operator to overcome what is a most tiresome operation with gold, in a manner acceptable and gratifying both in restoration and durability. The same can be said of bicuspids ; but now I am approaching treacherous ground, because the teeth are much used and more frequently decayed, offering a temptation for an esthetic operation such as can be obtained with a greater degree with this material.

A bicuspid is a small molar presenting large surfaces, and it has large contours with proportionately small supporting surfaces with a closely occluding antagonist that locks with every motion required for mastication. Add to this the great force brought to bear on an underlying substance like porcelain, which is stuck in position, and then you have an idea of the requirements necessary for a successful porcelain filling.

It may seem an exaggeration when I say, the larger the cavity the better the chance for success, and in many cases where I have supplied the whole palatine or buccal surface, they have given the greatest satisfaction, having had to replace but one in all these years. I account for that because of easy accessibility and the opportunity to change the form of the tooth, a better foundation giving the resistance of a large surface without leverage of an overhanging contour.

It is not my desire to discourage the filling of these teeth with porcelain, but rather to give you guidance that may save disappointments. I have had failures, but I am glad to say that my successes more than counterbalance them, otherwise I could not stand before you as an exponent of this work without fear of loss to my professional standing.

Passing from treacherous ground I will just say a few words about putting porcelain in molars and term it dangerous practice, because its use indiscriminately in these teeth has done more to discourage beginners than in any other portion of the mouth. Much that I

have said regarding bicuspids can be applied to molars, and yet under a few circumstances porcelain may be used to advantage, but in no case has its durability been equal to that which has been shown in other teeth.

The first objection is the difficulty in procuring sufficient space to obtain a correct mould of the cavity, and even when there are such opportunities, there is still the difficulty in holding the metal rigid until a perfect outline is secured. Succeeding in this there is still some uncertainty, as large surfaces in being fused frequently change form sufficient to cause a poor joint, which is a weak spot through loss of cement with wear and wash. I have been successful in making some very fair molar fillings and they have stood the wear for a number of years, but upon close observation I notice that the joints seem a trifle larger, which is caused by the constant pressure in mastication, breaking minute particles off the edges.

I have always used the high fusing body and my statements are based on the use of that material, and it has a record unapproached by any other of its kind. It is to be expected that in twelve years our materials would show improvement. The most notable is the variety of shades in porcelain and the uniformity of fusing, two great assistants. The improvement in appliances is the addition of electricity and the latest furnaces are simply perfect in form and practical working, making the possibility for perfect work within the reach of all.

During this time we have known how to do the work, but its ultimate success depended on one material and that was cement. It was our anchor and our only hope. Its failure means more than I, for one, care to contemplate, and while it is one of the materials that has not been improved in the life of this work and it is still deficient, yet I forgive it for the total good results. It has spoiled the beauty of our almost perfectly shaded inlay and caused much disappointment, but it has virtues that will probably never be equaled. I have used many that are good and those that had the greatest number of requisites as assistants to porcelain I used loyally and through their efficiency I am enabled to give you this paper.

In the last year or two I have added to my list, and if after a few years I find them equal to my standard I will give them all the credit due.

Any dentist practicing this branch of dentistry and having fair success will learn many advantages that will compensate for any losses that may arise through the acquirement of the knowledge and practice sufficient to insure confidence because tooth structure is supplied with a material approximating what has been lost. It strengthens poor teeth to a surprising degree—in fact, I have seen many cases where teeth have broken away, leaving the porcelain strong and intact, particularly in bicuspids. It is less tiresome to both patient and operator and allows a brief respite to the unavoidable exchange of vitated atmosphere to which unfortunately we are subjected in the practice of dentisty One of its greatest recommendations is the fact of being a perfect non-conductor, at least as near perfect as any filling can be.—*Items of Interest.*

